# Supplementary material for: Coevolutionary dynamics of cooperation, risk, and cost in collective risk games
Source: PLoS Comput Biol. 2026 Feb 20;22(2):e1013512. doi: 10.1371/journal.pcbi.1013512 (PMC12923141; doi:10.1371/journal.pcbi.1013512)
Supplement: S1 File — Figs A and B illustrate the robustness results of Model 1. Figures C through H display the theoretical and numerical results of Model 2. Table A summarizes the stability conditions of the equilibrium points in Model 2. (PDF) [file pcbi.1013512.s001.pdf]

Supplementary Information for Coevolutionary dynamics of cooperation, risk, and cost in collective risk games

Lichen Wang[1◑], Shijia Hua[1◑], Yuyuan Liu[1], Liang Zhang[1], Linjie Liu[1,2*], Attila Szolnoki[3],

1 College of Science, Northwest A & F University, Yangling, China
2 College of Economics & Management, Northwest A & F University, Yangling, China
3 Institute of Technical Physics and Materials Science, Centre for Energy Research, Budapest, Hungary

◑These authors contributed equally to this work.
* linjie140@126.com

Supporting information

In this Supporting Information, we provide the distribution of equilibrium points for Model 1 along with its stability analysis. Subsequently, we present a variant of Model 1 that introduces an additional feedback-evolving game framework, and we conduct a theoretical analysis of this modified system. Finally, we numerically investigate the effects of feedback speeds on evolutionary outcomes in both Model 1 and Model 2, while also examining the impact of several key model parameters on the basin of attraction of stable equilibrium points.

Labeling convention for equilibrium points

To improve clarity, we explicitly explain the labeling convention used throughout the manuscript. Since all phase portraits of the parameter cube are presented from a fixed viewing perspective, equilibrium points located on the faces are labeled according to their geometric positions. Specifically, the top face ($c = \alpha$) is denoted by “T” (Top), the bottom face ($c = \beta$) by “B” (Bottom), the left face ($r = 0$) by “L” (Left), the right face ($r = 1$) by “R” (Right), the front face ($x = 1$) by “F” (Front), and the back face ($x = 0$) by “K” (Back). For equilibrium points located on the edges, we use the lowercase labels “t” and “b” to indicate whether the point lies on the top edge ($c = \alpha$ and $r = 1$) or the bottom edge ($c = \beta$ and $r = 1$), respectively.

1 Model 1

We first analyze the equilibrium structure of the coupled system Eq. (10) and identify eight corner equilibrium points, namely $(0, 0, \alpha)$, $(0, 0, \beta)$, $(0, 1, \alpha)$, $(0, 1, \beta)$, $(1, 0, \alpha)$, $(1, 0, \beta)$, $(1, 1, \alpha)$, and $(1, 1, \beta)$. When $b\left(\frac{N-M}{N-1}\right)^{N-M}\left(\frac{M-1}{N-1}\right)^{M-1}\binom{N-1}{M-1} > \alpha$, the system admits two edge equilibrium points $(x_{1t}, 1, \alpha)$ and $(x_{2t}, 1, \alpha)$, where x_{1t} and x_{2t} are the two roots of the equation $b\binom{N-1}{M-1}x^{M-1}(1-x)^{N-M} = \alpha$ and satisfy $x_{1t} < x_{2t}$. Similarly, when $b\left(\frac{N-M}{N-1}\right)^{N-M}\left(\frac{M-1}{N-1}\right)^{M-1}\binom{N-1}{M-1} > \beta$, the system admits two edge equilibrium points $(x_{1b}, 1, \beta)$ and $(x_{2b}, 1, \beta)$, with x_{1b} and x_{2b} being the two roots of

$b\binom{N-1}{M-1}x^{M-1}(1-x)^{N-M}=\beta$ and satisfying $x_{1b}<x_{2b}$. Moreover, when $0<B_r<1$, the system admits a surface equilibrium point (x^*,B_r,β), where $x^*=\frac{\mu_1}{\mu_1+\mu_2}$ and $B_r=\frac{\beta}{b\binom{N-1}{M-1}x^{*M-1}(1-x^*)^{N-M}}$. When $\theta_3<\theta_2$ and $\beta<R_c<\alpha$, the system admits a surface equilibrium point $(R_x,1,R_c)$, where $R_x=\frac{\theta_1+\theta_3}{\theta_1+\theta_2}$ and $R_c=b\binom{N-1}{M-1}{R_x}^{M-1}(1-R_x)^{N-M}$. Moreover, when $0<T_r<1$, the system has a surface equilibrium point (x^*,T_r,α), where $T_r=\frac{\alpha}{b\binom{N-1}{M-1}x^{*M-1}(1-x^*)^{N-M}}$. Finally, when $0<r^*<1$ and $\beta<c^*<\alpha$, the system admits an interior equilibrium point (x^*,r^*,c^*), where $r^*=\frac{\theta_2\mu_1-\theta_1\mu_2+\theta_4(\mu_1+\mu_2)}{(\theta_3+\theta_4)(\mu_1+\mu_2)}$ and $c^*=\binom{N-1}{M-1}x^{*M-1}(1-x^*)^{N-M}r^*b$.

1.1 Stability analysis of equilibrium points

We next linearize the system and analyze the signs of the real parts of the eigenvalues of the Jacobian matrix evaluated at the equilibrium points to determine their stability [1–3]. When all eigenvalues of the Jacobian matrix evaluated at an equilibrium point have negative real parts, the equilibrium is stable. Conversely, if at least one eigenvalue has a positive real part, the equilibrium is unstable. For convenience, we define

$$\begin{cases} m(x,r,c)=x(1-x)\left[\dbinom{N-1}{M-1}x^{M-1}(1-x)^{N-M}rb-c\right], \\ n(x,r,c)=r(1-r)\left[\mu_1(1-x)-\mu_2 x\right], \\ p(x,r,c)=(\alpha-c)(c-\beta)\left[\theta_1(1-x)-\theta_2 x+\theta_3 r-\theta_4(1-r)\right]. \end{cases}$$

Accordingly, the Jacobian matrix of the system can be written as

$$J=\begin{bmatrix} \frac{\partial m(x,r,c)}{\partial x} & \frac{\partial m(x,r,c)}{\partial r} & \frac{\partial m(x,r,c)}{\partial c} \\ \frac{\partial n(x,r,c)}{\partial x} & \frac{\partial n(x,r,c)}{\partial r} & \frac{\partial n(x,r,c)}{\partial c} \\ \frac{\partial p(x,r,c)}{\partial x} & \frac{\partial p(x,r,c)}{\partial r} & \frac{\partial p(x,r,c)}{\partial c} \end{bmatrix},$$

where

$$\begin{cases} \dfrac{\partial m(x,r,c)}{\partial x}=\dbinom{N-1}{M-1}rbx^{M-1}(1-x)^{N-M}(M-Nx-x)+c(2x-1), \\ \dfrac{\partial m(x,r,c)}{\partial r}=\dbinom{N-1}{M-1}bx^{M}(1-x)^{N-M+1}, \\ \dfrac{\partial m(x,r,c)}{\partial c}=x(x-1), \\ \dfrac{\partial n(x,r,c)}{\partial x}=r(r-1)(\mu_1+\mu_2), \\ \dfrac{\partial n(x,r,c)}{\partial r}=(1-2r)[\mu_1(1-x)-\mu_2 x], \\ \dfrac{\partial n(x,r,c)}{\partial c}=0, \\ \dfrac{\partial p(x,r,c)}{\partial x}=(\alpha-c)(\beta-c)(\theta_1+\theta_2), \\ \dfrac{\partial p(x,r,c)}{\partial r}=(\alpha-c)(c-\beta)(\theta_3+\theta_4), \\ \dfrac{\partial p(x,r,c)}{\partial c}=(\alpha+\beta-2c)[\theta_1(1-x)-\theta_2 x+\theta_3 r-\theta_4(1-r)]. \end{cases}$$

The Jacobian matrices evaluated at the equilibrium points are given as follows: 44

$$J(0,0,\alpha) = \begin{bmatrix} -\alpha & 0 & 0 \\ 0 & \mu_1 & 0 \\ 0 & 0 & (\beta-\alpha)(\theta_1-\theta_4) \end{bmatrix},$$

45

$$J(0,0,\beta) = \begin{bmatrix} -\beta & 0 & 0 \\ 0 & \mu_1 & 0 \\ 0 & 0 & (\alpha-\beta)(\theta_1-\theta_4) \end{bmatrix},$$

46

$$J(0,1,\alpha) = \begin{bmatrix} -\alpha & 0 & 0 \\ 0 & -\mu_1 & 0 \\ 0 & 0 & (\beta-\alpha)(\theta_1+\theta_3) \end{bmatrix},$$

47

$$J(0,1,\beta) = \begin{bmatrix} -\beta & 0 & 0 \\ 0 & -\mu_1 & 0 \\ 0 & 0 & (\alpha-\beta)(\theta_1+\theta_3) \end{bmatrix},$$

48

$$J(1,0,\alpha) = \begin{bmatrix} \alpha & 0 & 0 \\ 0 & -\mu_2 & 0 \\ 0 & 0 & (\alpha-\beta)(\theta_2+\theta_4) \end{bmatrix},$$

49

$$J(1,0,\beta) = \begin{bmatrix} \beta & 0 & 0 \\ 0 & -\mu_2 & 0 \\ 0 & 0 & (\beta-\alpha)(\theta_2+\theta_4) \end{bmatrix},$$

50

$$J(1,1,\alpha) = \begin{bmatrix} \alpha & 0 & 0 \\ 0 & \mu_2 & 0 \\ 0 & 0 & (\alpha-\beta)(\theta_2-\theta_3) \end{bmatrix},$$

51

$$J(1,1,\beta) = \begin{bmatrix} \beta & 0 & 0 \\ 0 & \mu_2 & 0 \\ 0 & 0 & (\alpha-\beta)(\theta_3-\theta_2) \end{bmatrix},$$

$$J(x_t,1,\alpha) = \begin{bmatrix} t_1 & t_2 & t_3 \\ 0 & t_4 & 0 \\ 0 & 0 & t_5 \end{bmatrix},$$

where 52

$$\begin{cases} t_1 = \alpha\left(M - Nx_t - x_t\right) + \alpha\left(2x_t - 1\right), \\ t_2 = b\dbinom{N-1}{M-1} {x_t}^M \left(1 - x_t\right)^{N-M+1}, \\ t_3 = x_t\left(x_t - 1\right), \\ t_4 = -\left[\mu_1\left(1 - x_t\right) - \mu_2 x_t\right], \\ t_5 = (\beta - \alpha)\left[\theta_1\left(1 - x_t\right) - \theta_2 x_t + \theta_3\right], \end{cases}$$

Here, x_t denotes either of the two edge equilibrium solutions x_{1t} or x_{2t}. 53

$$J(x_b,1,\beta) = \begin{bmatrix} b_1 & b_2 & b_3 \\ 0 & b_4 & 0 \\ 0 & 0 & b_5 \end{bmatrix},$$

where

$$
\begin{cases}
b_1 = \beta\left(M - Nx_b - x_b\right) + \beta\left(2x_b - 1\right), \\
b_2 = b\dbinom{N-1}{M-1}{x_b}^{M}\left(1 - x_b\right)^{N-M+1}, \\
b_3 = x_b\left(x_b - 1\right), \\
b_4 = -\left[\mu_1\left(1 - x_b\right) - \mu_2 x_b\right], \\
b_5 = \left(\alpha - \beta\right)\left[\theta_1\left(1 - x_b\right) - \theta_2 x_b + \theta_3\right],
\end{cases}
$$

Here, x_b denotes either of the two edge equilibrium solutions x_{1b} or x_{2b}.

$$
J\left(x^*, B_r, \beta\right) = \begin{bmatrix} B_1 & B_2 & B_3 \\ B_4 & 0 & 0 \\ 0 & 0 & B_5 \end{bmatrix},
$$

where

$$
\begin{cases}
B_1 = \beta(M - Nx^* - x^*) + \beta(2x^* - 1), \\
B_2 = b\dbinom{N-1}{M-1}{x^*}^{M}(1 - x^*)^{N-M+1}, \\
B_3 = x^*(x^* - 1), \\
B_4 = B_r(B_r - 1)(\mu_1 + \mu_2), \\
B_5 = \left(\alpha - \beta\right)\left[\theta_1(1 - x^*) - \theta_2 x^* + \theta_3 B_r - \theta_4(1 - B_r)\right].
\end{cases}
$$

$$
J\left(x^*, T_r, \alpha\right) = \begin{bmatrix} T_1 & T_2 & T_3 \\ T_4 & 0 & 0 \\ 0 & 0 & T_5 \end{bmatrix},
$$

where

$$
\begin{cases}
T_1 = \alpha(M - Nx^* - x^*) + \alpha(2x^* - 1), \\
T_2 = b\dbinom{N-1}{M-1}{x^*}^{M}(1 - x^*)^{N-M+1}, \\
T_3 = x^*(x^* - 1), \\
T_4 = T_r(T_r - 1)(\mu_1 + \mu_2), \\
T_5 = \left(\beta - \alpha\right)\left[\theta_1(1 - x^*) - \theta_2 x^* + \theta_3 T_r - \theta_4(1 - T_r)\right].
\end{cases}
$$

$$
J\left(R_x, 1, R_c\right) = \begin{bmatrix} R_1 & R_2 & R_3 \\ 0 & R_4 & 0 \\ R_5 & R_6 & 0 \end{bmatrix},
$$

where

$$
\begin{cases}
R_1 = R_c(M - NR_x - R_x) + R_c(2R_x - 1), \\
R_2 = b\dbinom{N-1}{M-1}{R_x}^{M}(1 - R_x)^{N-M+1}, \\
R_3 = R_x(R_x - 1), \\
R_4 = -\left[\mu_1(1 - R_x) - \mu_2 R_x\right], \\
R_5 = \left(\alpha - R_c\right)\left(\beta - R_c\right)\left(\theta_1 + \theta_2\right), \\
R_6 = \left(\alpha - R_c\right)\left(R_c - \beta\right)\left(\theta_3 + \theta_4\right).
\end{cases}
$$

$$
J(x^*, r^*, c^*) = \begin{bmatrix} I_1 & I_2 & I_3 \\ I_4 & 0 & 0 \\ I_5 & I_6 & 0 \end{bmatrix},
$$

where

$$
\begin{cases}
I_1 = c^*(M - Nx^* - x^*) + c^*(2x^* - 1), \\
I_2 = b\binom{N-1}{M-1} x^{*M} (1 - x^*)^{N-M+1}, \\
I_3 = x^*(x^* - 1), \\
I_4 = r^*(r^* - 1)(\mu_1 + \mu_2), \\
I_5 = (\alpha - c^*)(\beta - c^*)(\theta_1 + \theta_2), \\
I_6 = (\alpha - c^*)(c^* - \beta)(\theta_3 + \theta_4).
\end{cases}
$$

Here, we present the stability analysis for all equilibrium points. Specifically, for the equilibrium points located at the corners and edges, the Jacobian matrix is triangular (or diagonal), so its eigenvalues are given by the diagonal elements; therefore, we use the diagonal elements to assess stability. For the surface and interior equilibrium points, we introduce the following lemma to evaluate their stability.

Lemma 1 *Consider the polynomial equation*

$$\lambda^n + a_1\lambda^{n-1} + a_2\lambda^{n-2} + \ldots + a_{n-1}\lambda + a_n = 0. \tag{A1}$$

The necessary condition for all roots of Eq. (A1) to have negative real parts is given by:

$$a_j > 0, \quad \forall j = 1, 2, \cdots, n. \tag{A2}$$

1.2 Stability of edge equilibrium points

For the edge equilibrium points, when $(x_{1t}, 1, \alpha)$ and $(x_{2t}, 1, \alpha)$ exist, the examination of the Jacobian matrix reveals the stability criteria for these equilibrium points:

$$
\begin{cases}
\alpha(M - Nx_t - x_t) + \alpha(2x_t - 1) < 0, \\
-[\mu_1(1 - x_t) - \mu_2 x_t] < 0, \\
(\beta - \alpha)[\theta_1(1 - x_t) - \theta_2 x_t + \theta_3] < 0.
\end{cases}
$$

The equivalent stability conditions obtained by computation are:

$$\frac{M-1}{N-1} < x_t < \min\left\{\frac{\mu_1}{\mu_1 + \mu_2}, \frac{\theta_1 + \theta_3}{\theta_1 + \theta_2}\right\}.$$

Following a similar analysis, the stability condition for the edge equilibria $(x_{1b}, 1, \beta)$ and $(x_{2b}, 1, \beta)$ is

$$\max\left\{\frac{\theta_1 + \theta_3}{\theta_1 + \theta_2}, \frac{M-1}{N-1}\right\} < x_b < \frac{\mu_1}{\mu_1 + \mu_2}.$$

1.3 Stability of surface equilibrium points

When the surface equilibrium point $(R_x, 1, R_c)$ exists, the characteristic polynomial of $J(R_x, 1, R_c)$ is given by

$$\lambda^3 - (R_4 + R_1)\lambda^2 + (R_1R_4 - R_3R_5)\lambda + R_3R_4R_5 = 0.$$

It is also clear that $R_3 < 0$ and $R_5 < 0$. Using Lemma 1, suppose that all eigenvalues of the characteristic polynomial have negative real parts. Then all coefficients of the

polynomial must be positive. However, since $R_3 < 0$ and $R_5 < 0$, we have $R_3R_5 > 0$, and the positivity of the constant term $R_3R_4R_5$ requires $R_4 > 0$. This in turn implies that $R_1 > 0$ in order for the coefficient $R_1R_4 - R_3R_5$ to be positive. Consequently, $R_1 + R_4 > 0$, which makes the coefficient of λ^2 negative. This contradicts the necessary condition in Lemma 1. Therefore, the surface equilibrium point $(R_x, 1, R_c)$ is unstable.

When the surface equilibrium point (x^*, T_r, α) exists, the characteristic polynomial of $J(x^*, T_r, \alpha)$ is given by

$$(T_5 - \lambda)\left(-T_2T_4 - T_1\lambda + \lambda^2\right) = 0.$$

We note that one eigenvalue of $J(x^*, T_r, \alpha)$ is $\lambda_1 = T_5$. Let λ_2 and λ_3 be the other two distinct eigenvalues from λ_1. Then we have $\lambda_2 + \lambda_3 = T_1$ and $\lambda_2\lambda_3 = -T_2T_4$. Since $T_2 > 0$ and $T_4 < 0$, we conclude that the surface equilibrium point (x^*, T_r, α) is stable when $T_5 < 0$ and $T_1 < 0$.

When the surface equilibrium point (x^*, B_r, β) exists, the characteristic polynomial of $J(x^*, B_r, \beta)$ is given by

$$(B_5 - \lambda)\left(-B_2B_4 - B_1\lambda + \lambda^2\right) = 0.$$

Similarly to the analysis for the surface equilibrium point (x^*, T_r, α), our findings suggest that when $B_1 < 0$ and $B_5 < 0$, the surface equilibrium point (x^*, B_r, β) is stable.

1.4 Stability of interior equilibrium points

Assuming the interior equilibrium point (x^*, r^*, c^*) exists, the corresponding characteristic polynomial of $J(x^*, r^*, c^*)$ can be expressed as follows:

$$\lambda^3 - I_1\lambda^2 - (I_2I_4 + I_3I_5)\lambda - I_3I_4I_6 = 0. \tag{A3}$$

Upon evaluation, we observe that $I_2 > 0$, $I_3 < 0$, $I_5 < 0$, and $I_6 > 0$. Referring to Lemma 1, if the eigenvalues of this characteristic polynomial all possess negative real parts, it implies that $I_4 > 0$. However, in this scenario, $I_2I_4 + I_3I_5 > 0$ creates a contradiction. Consequently, we claim that the interior equilibrium point (x^*, r^*, c^*) is unstable.

The theoretical results derived above are numerically confirmed in the main text. It should be noted that, for analytical convenience, the feedback speeds ε_1 and ε_2 were previously set to 1. Next, we investigate the influence of feedback speeds on the evolutionary dynamics, using the stable surface equilibrium point (x^*, B_r, β) as a representative case. Fig A (top row) shows that, with ε_2 fixed at 2, the stability of (x^*, B_r, β) is maintained for different values of ε_1, while the rate of convergence to this equilibrium varies. Similarly, the bottom row shows that, with ε_1 fixed at 2, varying ε_2 does not alter the stability of the equilibrium but affects the convergence speed.

The coupled system exhibits monostability, bistability, and tristability. As shown in Fig B, we systematically investigate how the key feedback strengths θ_1, θ_3, and μ_1 govern both the type of stable equilibria and the sizes of their basins of attraction. For low θ_3, the system is bistable between the edge equilibrium $(x_{2b}, 1, \beta)$ and the corner equilibrium $(0, 1, \alpha)$, with the former possessing a larger basin of attraction. As θ_3 increases, a transition occurs: $(x_{2b}, 1, \beta)$ loses stability, while the high cost edge equilibrium $(x_{2t}, 1, \alpha)$ becomes stable and attains a larger basin of attraction. This implies that when risk strongly inflates cooperation cost, sustaining cooperation requires bearing the maximal cost α, yet this state remains accessible under appropriate initial conditions. The influence of θ_1 is modulated by θ_3. When θ_3 is small, θ_1 affects

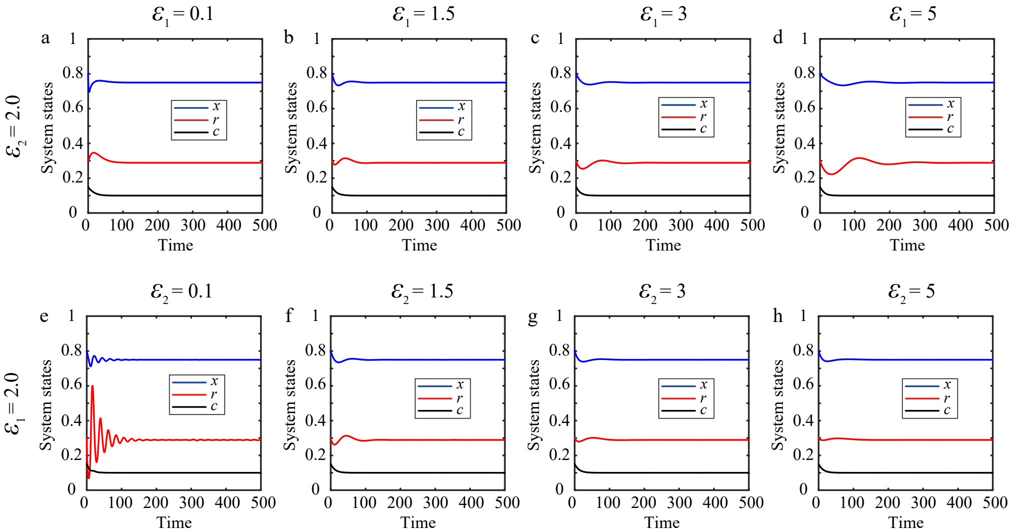

Fig A. Impact of feedback speeds on the evolutionary dynamics when the system converges to a stable surface equilibrium point (x^*, B_r, β). The top (bottom) row shows the effect of the feedback speed ε_1 (ε_2) on the time evolution of the system state. Parameters are $N = 8$, $M = 5$, $\mu_1 = 0.9$, $\mu_2 = 0.3$, $\theta_1 = 0.5$, $\theta_2 = 0.5$, $\theta_3 = 0.8$, $\theta_4 = 0.6$, $b = 2$, $\beta = 0.1$, and $\alpha = 0.3$ in all panels.

the basins of attraction in a manner similar to the θ_3 regime; however, under strong risk-driven cost inflation (large θ_3), θ_1 has a negligible impact. Regarding μ_1, small values lead only to the tragic equilibrium $(0, 1, \alpha)$. Increasing μ_1 induces two successive tristable phases: first among $(0, 1, \alpha)$, (x^*, B_r, β), and (x^*, T_r, α); then among $(0, 1, \alpha)$, (x^*, B_r, β), and $(x_{2t}, 1, \alpha)$. At even larger μ_1, the system returns to bistability between $(0, 1, \alpha)$ and the edge equilibrium $(x_{2t}, 1, \alpha)$, with the latter possessing a larger basin of attraction. Finally, increases in either θ_1 or θ_3 systematically enlarge the basin of attraction of the high cost surface equilibrium (x^*, T_r, α) while shrinking that of the low cost one (x^*, B_r, β).

2 Model 2

In the previous model, we assumed that changes in the cost of cooperation did not affect the risk level. Here, we extend the model by introducing an additional feedback mechanism in which an increase in the cost of cooperation reduces the risk level, whereas a decrease in the cost of cooperation increases the risk (Fig C). The coupled game model with multiple feedback mechanisms is given by

$$\begin{cases} \dot{x} = x(1-x)\left[\binom{N-1}{M-1}x^{M-1}(1-x)^{N-M}rb - c\right], \\ \dot{r} = r(1-r)\left[\mu_1(1-x) - \mu_2 x - \mu_3(c-\beta) + \mu_4(\alpha - c)\right], \\ \dot{c} = (\alpha - c)(c-\beta)\left[\theta_1(1-x) - \theta_2 x + \theta_3 r - \theta_4(1-r)\right], \end{cases} \tag{A4}$$

where μ_3 and μ_4 represent the coefficients quantifying how an increase and a decrease in the cost of cooperation reduce and increase the level of risk, respectively.

We first analyze the distribution of equilibria. For clarity, we use the same notation in Model 2 as in Model 1. Based on Eq. (A4), we identify the corner equilibrium points of the system as: $(0, 0, \alpha)$, $(0, 0, \beta)$, $(0, 1, \alpha)$, $(0, 1, \beta)$, $(1, 0, \alpha)$, $(1, 0, \beta)$, $(1, 1, \alpha)$, and $(1, 1, \beta)$.

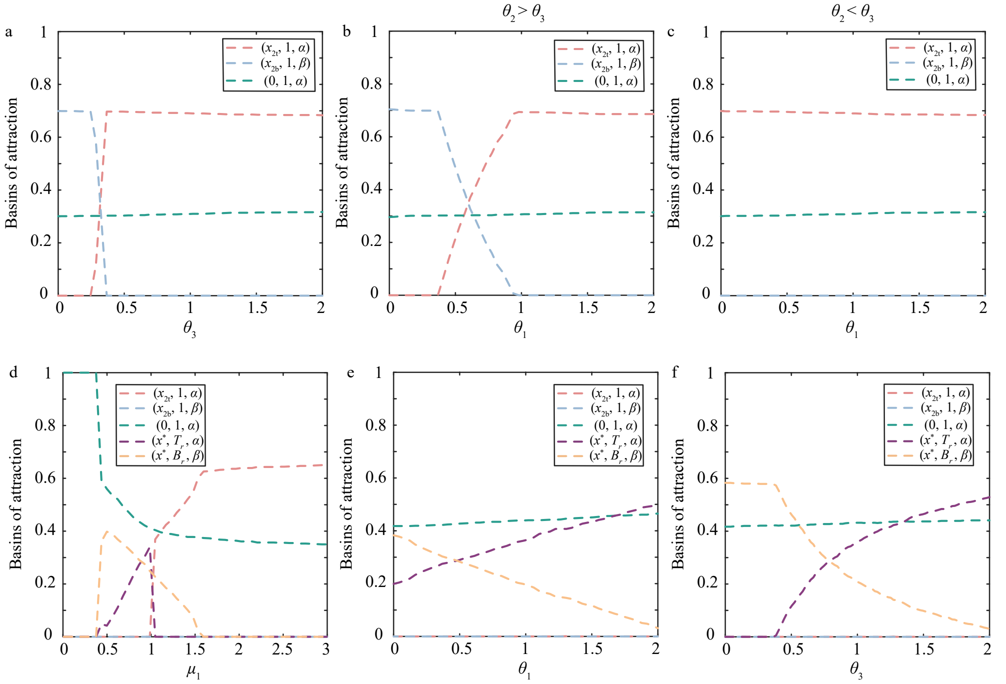

Fig B. Effects of model parameters on the basins of attraction of stable equilibria in the coupled system. Parameters are $\mu_1 = 2.5$, $\theta_1 = 0.5$, $\theta_2 = 0.5$ in panel a; $\mu_1 = 2.5$, $\theta_2 = 0.5$, $\theta_3 = 0.3$ in panel b; $\mu_1 = 2.5$, $\theta_2 = 0.3$, $\theta_3 = 0.5$ in panel c; $\theta_1 = 0.5$, $\theta_2 = 0.5$, $\theta_3 = 0.8$ in panel d; $\mu_1 = 0.9$, $\theta_2 = 0.5$, $\theta_3 = 0.8$ in panel e; $\mu_1 = 0.9$, $\theta_1 = 0.5$, $\theta_2 = 0.5$ in panel f. The remaining parameters $N = 8$, $M = 5$, $\mu_2 = 0.3$, $\theta_4 = 0.6$, $b = 2$, $\beta = 0.1$, and $\alpha = 0.3$ are fixed across all panels.

For the edge equilibrium points, when $b\left(\frac{N-M}{N-1}\right)^{N-M}\left(\frac{M-1}{N-1}\right)^{M-1}\binom{N-1}{M-1} > \alpha$, the system admits two edge equilibrium points, $(x_{1t}, 1, \alpha)$ and $(x_{2t}, 1, \alpha)$. Here, x_{1t} and x_{2t} are the two distinct roots of the equation $b\binom{N-1}{M-1}x^{M-1}(1-x)^{N-M} = \alpha$, satisfying $x_{1t} < x_{2t}$. Similarly, when $b\left(\frac{N-M}{N-1}\right)^{N-M}\left(\frac{M-1}{N-1}\right)^{M-1}\binom{N-1}{M-1} > \beta$, the system admits two edge equilibrium points, $(x_{1b}, 1, \beta)$ and $(x_{2b}, 1, \beta)$, where x_{1b} and x_{2b} are the two distinct roots of the equation $b\binom{N-1}{M-1}x^{M-1}(1-x)^{N-M} = \beta$, satisfying $x_{1b} < x_{2b}$.

For the surface equilibrium points, when $0 < K_r < 1$ and $\beta < K_c < \alpha$, the system has a surface equilibrium point $(0, K_r, K_c)$, where $K_r = \frac{\theta_4-\theta_1}{\theta_3+\theta_4}$ and $K_c = \frac{\mu_1+\mu_3\beta+\mu_4\alpha}{\mu_3+\mu_4}$. When $0 < R_x < 1$ and $\beta < R_c < \alpha$, the system has a surface equilibrium point $(R_x, 1, R_c)$, where $R_x = \frac{\theta_1+\theta_3}{\theta_1+\theta_2}$ and $R_c = \binom{N-1}{M-1}R_x^{M-1}(1-R_x)^{N-M}b$. When $0 < F_r < 1$ and $\beta < F_c < \alpha$, the system has a surface equilibrium point $(1, F_r, F_c)$, where $F_r = \frac{\theta_2+\theta_4}{\theta_3+\theta_4}$ and $F_c = \frac{-\mu_2+\mu_3\beta+\mu_4\alpha}{\mu_3+\mu_4}$. When $0 < T_x < 1$ and $0 < T_r < 1$, the system has a surface equilibrium point (T_x, T_r, α), where $T_x = \frac{\mu_1-\mu_3(\alpha-\beta)}{\mu_1+\mu_2}$ and $T_r = \frac{\alpha}{b\binom{N-1}{M-1}T_x{}^{M-1}(1-T_x)^{N-M}}$. When $0 < B_x < 1$ and $0 < B_r < 1$, the system has a surface equilibrium point (B_x, B_r, β), where $B_x = \frac{\mu_1+\mu_4(\alpha-\beta)}{\mu_1+\mu_2}$ and $B_r = \frac{\beta}{b\binom{N-1}{M-1}B_x{}^{M-1}(1-B_x)^{N-M}}$.

For the interior equilibrium points, we consider solutions to the following equations within the ranges $x \in (0,1)$, $r \in (0,1)$, and $c \in (\beta, \alpha)$. Without loss of generality, we

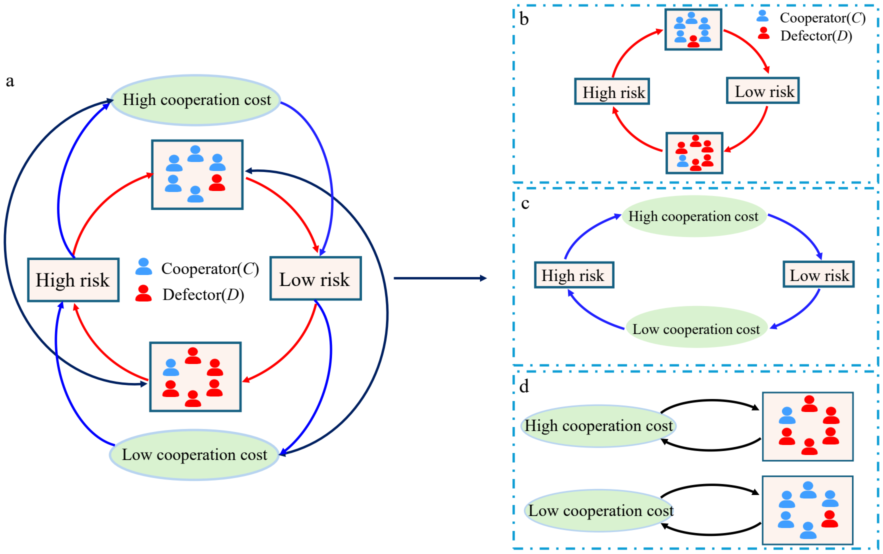

Fig C. Schematic illustration of Model 2. a: The collective-risk social dilemma game involves complex feedback relationships among the cooperation cost, group state, and risk level. b: Feedback between the group state and collective risk is illustrated. c: Feedback between collective risk and the cost of cooperation. d: High cooperation cost promotes defection within the group, and the resulting increase in defection further raises the cooperation cost. Conversely, low cooperation cost facilitates the spread of cooperation, which in turn reduces the cooperation cost further.

assume that the solution can be represented as (x^*, r^*, c^*). The equations are as follows: 163

$$\begin{cases} \binom{N-1}{M-1} x^{M-1}(1-x)^{N-M} rb - c = 0, \\ \mu_1(1-x) - \mu_2 x - \mu_3(c-\beta) + \mu_4(\alpha - c) = 0, \\ \theta_1(1-x) - \theta_2 x + \theta_3 r - \theta_4(1-r) = 0. \end{cases}$$

Here, we let 164

$$\begin{cases} m(x,r,c) = x(1-x)\left[\binom{N-1}{M-1} x^{M-1}(1-x)^{N-M} rb - c\right], \\ n(x,r,c) = r(1-r)\left[\mu_1(1-x) - \mu_2 x - \mu_3(c-\beta) + \mu_4(\alpha-c)\right], \\ p(x,r,c) = (\alpha-c)(c-\beta)\left[\theta_1(1-x) - \theta_2 x + \theta_3 r - \theta_4(1-r)\right]. \end{cases}$$

The Jacobian matrix of the system is given by: 165

$$J = \begin{bmatrix} \frac{\partial m(x,r,c)}{\partial x} & \frac{\partial m(x,r,c)}{\partial r} & \frac{\partial m(x,r,c)}{\partial c} \\ \frac{\partial n(x,r,c)}{\partial x} & \frac{\partial n(x,r,c)}{\partial r} & \frac{\partial n(x,r,c)}{\partial c} \\ \frac{\partial p(x,r,c)}{\partial x} & \frac{\partial p(x,r,c)}{\partial r} & \frac{\partial p(x,r,c)}{\partial c} \end{bmatrix},$$

where

$$\begin{cases} \dfrac{\partial m(x,r,c)}{\partial x} = \dbinom{N-1}{M-1} rbx^{M-1}(1-x)^{N-M}(M-Nx-x)+c(2x-1), \\ \dfrac{\partial m(x,r,c)}{\partial r} = \dbinom{N-1}{M-1} bx^{M}(1-x)^{N-M+1}, \\ \dfrac{\partial m(x,r,c)}{\partial c} = x(x-1), \\ \dfrac{\partial n(x,r,c)}{\partial x} = r(r-1)(\mu_1+\mu_2), \\ \dfrac{\partial n(x,r,c)}{\partial r} = (1-2r)[\mu_1(1-x)-\mu_2 x-\mu_3(c-\beta)+\mu_4(\alpha-c)], \\ \dfrac{\partial n(x,r,c)}{\partial c} = r(r-1)(\mu_3+\mu_4), \\ \dfrac{\partial p(x,r,c)}{\partial x} = (\alpha-c)(\beta-c)(\theta_1+\theta_2), \\ \dfrac{\partial p(x,r,c)}{\partial r} = (\alpha-c)(c-\beta)(\theta_3+\theta_4), \\ \dfrac{\partial p(x,r,c)}{\partial c} = (\alpha+\beta-2c)[\theta_1(1-x)-\theta_2 x+\theta_3 r-\theta_4(1-r)]. \end{cases}$$

The specific form of the matrix at the corner equilibrium points is as follows:

$$J(0,0,\alpha) = \begin{bmatrix} -\alpha & 0 & 0 \\ 0 & \mu_1-(\alpha-\beta)\mu_3 & 0 \\ 0 & 0 & (\beta-\alpha)(\theta_1-\theta_4) \end{bmatrix},$$

$$J(0,0,\beta) = \begin{bmatrix} -\beta & 0 & 0 \\ 0 & \mu_1+(\alpha-\beta)\mu_4 & 0 \\ 0 & 0 & (\alpha-\beta)(\theta_1-\theta_4) \end{bmatrix},$$

$$J(0,1,\alpha) = \begin{bmatrix} -\alpha & 0 & 0 \\ 0 & -\mu_1+(\alpha-\beta)\mu_3 & 0 \\ 0 & 0 & (\beta-\alpha)(\theta_1+\theta_3) \end{bmatrix},$$

$$J(0,1,\beta) = \begin{bmatrix} -\beta & 0 & 0 \\ 0 & -\mu_1-(\alpha-\beta)\mu_4 & 0 \\ 0 & 0 & (\alpha-\beta)(\theta_1+\theta_3) \end{bmatrix},$$

$$J(1,0,\alpha) = \begin{bmatrix} \alpha & 0 & 0 \\ 0 & -\mu_2-(\alpha-\beta)\mu_3 & 0 \\ 0 & 0 & (\alpha-\beta)(\theta_2+\theta_4) \end{bmatrix},$$

$$J(1,0,\beta) = \begin{bmatrix} \beta & 0 & 0 \\ 0 & -\mu_2+(\alpha-\beta)\mu_4 & 0 \\ 0 & 0 & (\beta-\alpha)(\theta_2+\theta_4) \end{bmatrix},$$

$$J(1,1,\alpha) = \begin{bmatrix} \alpha & 0 & 0 \\ 0 & \mu_2+(\alpha-\beta)\mu_3 & 0 \\ 0 & 0 & (\alpha-\beta)(\theta_2-\theta_3) \end{bmatrix},$$

$$J(1,1,\beta) = \begin{bmatrix} \beta & 0 & 0 \\ 0 & \mu_2-(\alpha-\beta)\mu_4 & 0 \\ 0 & 0 & (\alpha-\beta)(\theta_3-\theta_2) \end{bmatrix}.$$

The specific form at the edge equilibrium point is as follows:

$$J(x_t, 1, \alpha) = \begin{bmatrix} t_1 & t_2 & t_3 \\ 0 & t_4 & 0 \\ 0 & 0 & t_5 \end{bmatrix},$$

where

$$\begin{cases} t_1 = \alpha\left(M - Nx_t - x_t\right) + \alpha\left(2x_t - 1\right), \\ t_2 = b\binom{N-1}{M-1} x_t^M (1 - x_t)^{N-M+1}, \\ t_3 = x_t\left(x_t - 1\right), \\ t_4 = -\left[\mu_1\left(1 - x_t\right) - \mu_2 x_t - \mu_3(\alpha - \beta)\right], \\ t_5 = (\beta - \alpha)\left[\theta_1\left(1 - x_t\right) - \theta_2 x_t + \theta_3\right], \end{cases}$$

Here, x_t denotes either of the two edge equilibrium solutions x_{1t} or x_{2t}.

$$J(x_b, 1, \beta) = \begin{bmatrix} b_1 & b_2 & b_3 \\ 0 & b_4 & 0 \\ 0 & 0 & b_5 \end{bmatrix},$$

where

$$\begin{cases} b_1 = \beta\left(M - Nx_b - x_b\right) + \beta\left(2x_b - 1\right), \\ b_2 = b\binom{N-1}{M-1} x_b^M (1 - x_b)^{N-M+1}, \\ b_3 = x_b\left(x_b - 1\right), \\ b_4 = -\left[\mu_1\left(1 - x_b\right) - \mu_2 x_b + \mu_4(\alpha - \beta)\right], \\ b_5 = (\alpha - \beta)\left[\theta_1\left(1 - x_b\right) - \theta_2 x_b + \theta_3\right], \end{cases}$$

Here, x_b denotes either of the two edge equilibrium solutions x_{1b} or x_{2b}. The specific form at the surface equilibrium point is as follows:

$$J\left(0, K_r, K_c\right) = \begin{bmatrix} K_1 & 0 & 0 \\ K_2 & 0 & K_3 \\ K_4 & K_5 & 0 \end{bmatrix},$$

where

$$\begin{cases} K_1 = -K_c, \\ K_2 = K_r(K_r - 1)(\mu_1 + \mu_2), \\ K_3 = K_r(K_r - 1)(\mu_3 + \mu_4), \\ K_4 = (\alpha - K_c)(\beta - K_c)(\theta_1 + \theta_2), \\ K_5 = (\alpha - K_c)(K_c - \beta)(\theta_3 + \theta_4). \end{cases}$$

$$J\left(R_x, 1, R_c\right) = \begin{bmatrix} R_1 & R_2 & R_3 \\ 0 & R_4 & 0 \\ R_5 & R_6 & 0 \end{bmatrix},$$

where

$$
\begin{cases}
R_1 = R_c(M - NR_x - R_x) + R_c(2R_x - 1), \\
R_2 = b\dbinom{N-1}{M-1}{R_x}^M(1 - R_x)^{N-M+1}, \\
R_3 = R_x(R_x - 1), \\
R_4 = -\left[\mu_1(1 - R_x) - \mu_2 R_x - \mu_3(R_c - \beta) + \mu_4(\alpha - R_c)\right], \\
R_5 = (\alpha - R_c)(\beta - R_c)(\theta_1 + \theta_2), \\
R_6 = (\alpha - R_c)(R_c - \beta)(\theta_3 + \theta_4).
\end{cases}
$$

$$
J\left(1, F_r, F_c\right) = \begin{bmatrix} F_1 & 0 & 0 \\ F_2 & 0 & F_3 \\ F_4 & F_5 & 0 \end{bmatrix},
$$

where

$$
\begin{cases}
F_1 = F_c, \\
F_2 = F_r(F_r - 1)(\mu_1 + \mu_2), \\
F_3 = F_r(F_r - 1)(\mu_3 + \mu_4), \\
F_4 = (\alpha - F_c)(\beta - F_c)(\theta_1 + \theta_2), \\
F_5 = (\alpha - F_c)(F_c - \beta)(\theta_3 + \theta_4).
\end{cases}
$$

$$
J\left(T_x, T_r, \alpha\right) = \begin{bmatrix} T_1 & T_2 & T_3 \\ T_4 & 0 & T_5 \\ 0 & 0 & T_6 \end{bmatrix},
$$

where

$$
\begin{cases}
T_1 = \alpha(M - NT_x - T_x) + \alpha(2T_x - 1), \\
T_2 = b\dbinom{N-1}{M-1}{T_x}^M(1 - T_x)^{N-M+1}, \\
T_3 = T_x(T_x - 1), \\
T_4 = T_r(T_r - 1)(\mu_1 + \mu_2), \\
T_5 = T_r(T_r - 1)(\mu_3 + \mu_4), \\
T_6 = (\beta - \alpha)\left[\theta_1(1 - T_x) - \theta_2 T_x + \theta_3 T_r - \theta_4(1 - T_r)\right].
\end{cases}
$$

$$
J\left(B_x, B_r, \beta\right) = \begin{bmatrix} B_1 & B_2 & B_3 \\ B_4 & 0 & B_5 \\ 0 & 0 & B_6 \end{bmatrix},
$$

where

$$
\begin{cases}
B_1 = \beta(M - NB_x - B_x) + \beta(2B_x - 1), \\
B_2 = b\dbinom{N-1}{M-1}{B_x}^M(1 - B_x)^{N-M+1}, \\
B_3 = B_x(B_x - 1), \\
B_4 = B_r(B_r - 1)(\mu_1 + \mu_2), \\
B_5 = B_r(B_r - 1)(\mu_3 + \mu_4), \\
B_6 = (\alpha - \beta)\left[\theta_1(1 - B_x) - \theta_2 B_x + \theta_3 B_r - \theta_4(1 - B_r)\right].
\end{cases}
$$

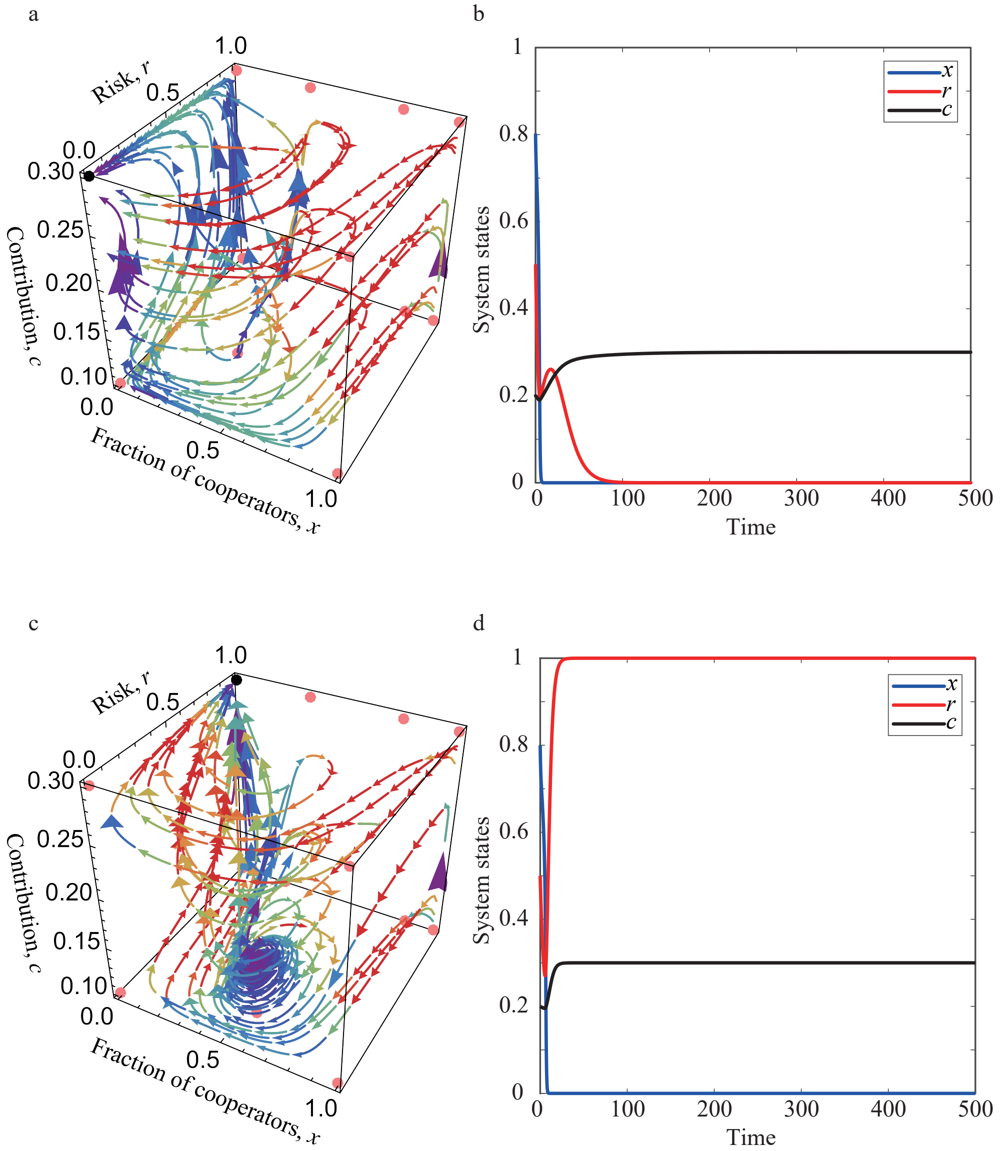

Fig D. The replicator dynamics of the coupled system when it exhibits a monostable result. Panels a and c show the phase flow in $x - r - c$ variable space. Red points represent unstable equilibrium points, while black points represent stable equilibrium points. The color of the curves represents the magnitude of the gradient. Panels b and d present the time evolution of coevolving variables. Parameters are $\mu_1 = 0.1$, $\mu_3 = 1$, $\mu_4 = 0.5$, $\theta_1 = 0.6$ in panels a and b; $\mu_1 = 0.4$, $\mu_3 = 0.5$, $\mu_4 = 0.5$, $\theta_1 = 1$ in panels c and d. The remaining parameters $N = 8$, $M = 5$, $\mu_2 = 0.5$, $\theta_2 = 0.5$, $\theta_3 = 0.6$, $\theta_4 = 0.5$, $b = 2$, $\beta = 0.1$, and $\alpha = 0.3$ are fixed in all panels.

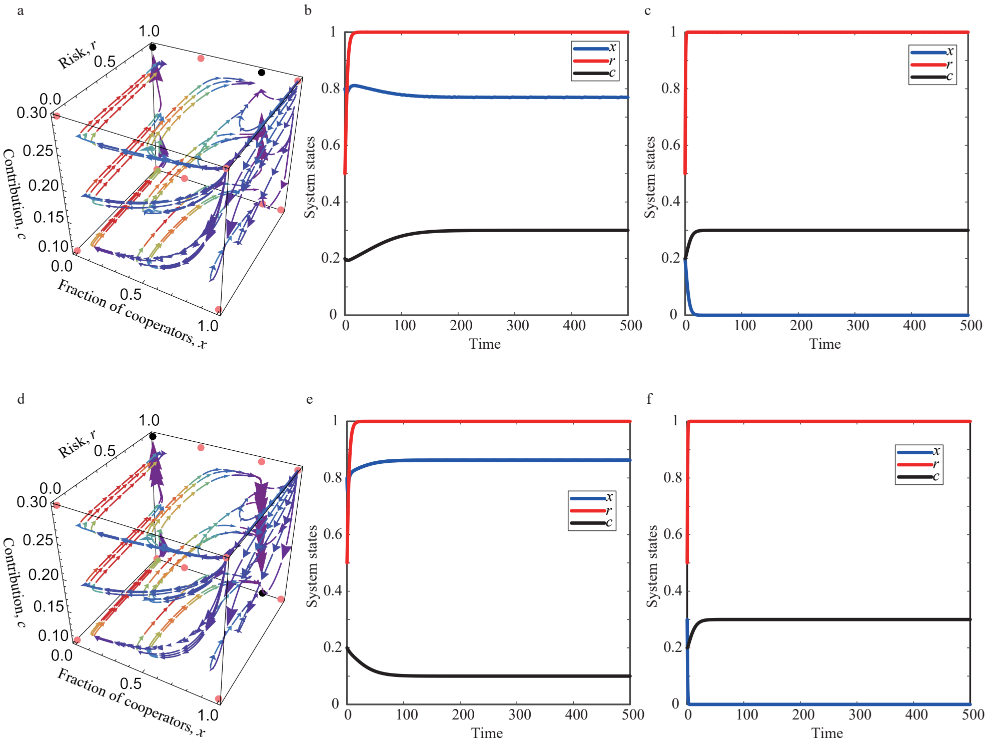

Fig E. The replicator dynamics of the coupled system when it exhibits stable edge equilibrium points. Panels a and d show the phase flow in $x - r - c$ space. Panels b, c, e, and f present the time evolution of coevolving variables. Parameter $\theta_3 = 0.4$ in panels a, b, and c; $\theta_3 = 0.1$ in panels d, e, and f. The remaining parameters $N = 8$, $M = 5$, $\mu_1 = 2.5$, $\mu_2 = 0.2$, $\mu_3 = 0.5$, $\mu_4 = 0.5$, $\theta_1 = 0.6$, $\theta_2 = 0.5$, $\theta_4 = 0.5$, $b = 2$, $\beta = 0.1$, and $\alpha = 0.3$ are fixed for all panels.

2.1 Stability Analysis

The stability of the corner equilibria is determined by the eigenvalues of their Jacobian matrices. When $\mu_1 < (\alpha - \beta)\mu_3$ and $\theta_1 > \theta_4$, the equilibrium point $(0, 0, \alpha)$ is stable, whereas when $\mu_1 > (\alpha - \beta)\mu_3$, the equilibrium point $(0, 1, \alpha)$ is stable. All other corner equilibria are unstable. Numerical examples confirm these theoretical results. As shown in Fig Da, for this specific parameter set, the system admits 13 equilibrium points, among which only $(0, 0, \alpha)$ is stable. This implies that, as a long-run evolutionary outcome, in a risk-free environment, no individual would choose to incur the highest cooperation cost to become a cooperator. The time evolution of the system state also confirms this result (Fig Db). In Fig Dc and Dd, we present another monostable result. For all initial points in the phase space, the system trajectories ultimately converge to point $(0, 1, \alpha)$. This state corresponds to the tragedy of the commons under maximal risk ($r = 1$): the high cooperation cost completely suppresses cooperation, resulting in universal defection and inevitable collective loss.

We analyze the stability properties of the equilibrium points located on the edges of the phase space. When $(x_{1t}, 1, \alpha)$ and $(x_{2t}, 1, \alpha)$ exist, $(x_{1t}, 1, \alpha)$ is unstable, since $x_{1t} < \frac{M-1}{N-1}$. In contrast, $(x_{2t}, 1, \alpha)$ is stable if $x_{2t} < \min\left\{\frac{\mu_1 - \mu_3(\alpha-\beta)}{\mu_1+\mu_2}, \frac{\theta_1+\theta_3}{\theta_1+\theta_2}\right\}$. Similarly, for the equilibria $(x_{1b}, 1, \beta)$ and $(x_{2b}, 1, \beta)$, $(x_{1b}, 1, \beta)$ is unstable, since $x_{1b} < \frac{M-1}{N-1}$, while $(x_{2b}, 1, \beta)$ is stable when $\frac{\theta_1+\theta_3}{\theta_1+\theta_2} < x_{2b} < \frac{\mu_1+\mu_4(\alpha-\beta)}{\mu_1+\mu_2}$. Furthermore, we provide numerical examples to validate the aforementioned theoretical analysis results

(Fig E). As shown in Fig Ea, the coupled system exhibits bistability, where the system trajectories converge to either the corner equilibrium point $(0, 1, \alpha)$ or the edge equilibrium point $(x_{2t}, 1, \alpha)$, depending on the initial conditions. The corner state represents the tragedy of the commons (full defection), whereas the edge state corresponds to a high-cooperation regime sustained at the maximal cost α under high risk ($r = 1$). This is illustrated by the sample time evolutions in Fig Eb and Ec. Fig Ed presents another bistable regime. In this regime, the system has two stable attractors: the tragic corner equilibrium $(0, 1, \alpha)$ and the cooperative edge equilibrium $(x_{2b}, 1, \beta)$. Consequently, the population may evolve either to full defection or to a state where a high level of cooperation is sustained at the minimal cost β, despite the prevailing high risk ($r = 1$). The time evolution in Fig Ee and Ef confirms the convergence to these two distinct outcomes.

We analyze the stability properties of the surface equilibrium points as follows. When the equilibrium point $(0, K_r, K_c)$ exists, we examine its stability on the plane $x = 0$. Note that when $x = 0$, system (A4) reduces to the following form:

$$\begin{cases} \dot{r} = r(1-r)\left[\mu_1 - \mu_3(c-\beta) + \mu_4(\alpha - c)\right], \\ \dot{c} = (\alpha - c)(c - \beta)\left[\theta_1 + \theta_3 r - \theta_4(1-r)\right]. \end{cases} \tag{A5}$$

By applying the transformations $\overline{r} = r - K_r$ and $\overline{c} = c - K_c$, system (A5) is transformed into:

$$\begin{cases} \dot{\overline{r}} = (\overline{r} + K_r)(1 - \overline{r} - K_r)\left[\mu_1 - \mu_3(\overline{c} + K_c - \beta) + \mu_4(\alpha - \overline{c} - K_c)\right], \\ \dot{\overline{c}} = (\alpha - \overline{c} - K_c)(\overline{c} + K_c - \beta)\left[\theta_1 + \theta_3(\overline{r} + K_r) - \theta_4(1 - \overline{r} - K_r)\right]. \end{cases} \tag{A6}$$

Thus, $(0, 0)$ is an equilibrium point of system (A6). A first integral of the system can be obtained as follows:

$$\frac{-P \ln|\bar{c} + K_c - \alpha| + Q \ln|\bar{c} + K_c - \beta|}{\alpha - \beta} + A \ln|1 - K_r - \bar{r}| + B \ln|K_r + \bar{r}| = K,$$

where K is a constant, $A = \theta_1 + \theta_3$, $B = \theta_4 - \theta_1$, $P = \mu_1 + (\beta - \alpha)\mu_3$, and $Q = \mu_1 + (\alpha - \beta)\mu_4$. Since the above equation is continuous in the small neighborhood around the origin $(0, 0)$, it follows that $(0, K_r, K_c)$ is a center in the surface defined by $x = 0$ [1].

When the surface equilibrium point $(R_x, 1, R_c)$ exists, the characteristic polynomial of $J\,(R_x, 1, R_c)$ can be written as

$$(\lambda - R_4)(\lambda^2 - R_1\lambda - R_3R_5) = 0.$$

Note that $R_3 < 0$ and $R_5 < 0$. Let λ_2 and λ_3 denote the two solutions of the equation $\lambda^2 - R_1\lambda - R_3R_5 = 0$, and observe that $\lambda_2\lambda_3 < 0$. Therefore, the surface equilibrium point $(R_x, 1, R_c)$ is unstable.

When the surface equilibrium point $(1, F_r, F_c)$ exists, the characteristic polynomial of $J\,(1, F_r, F_c)$ can be expressed as

$$(\lambda - F_1)(\lambda^2 - F_3F_5) = 0.$$

Since $F_1 > 0$, we conclude that the surface equilibrium point $(1, F_r, F_c)$ is unstable.

When the surface equilibrium point (T_x, T_r, α) exists, the characteristic polynomial of $J\,(T_x, T_r, \alpha)$ can be expressed as

$$(\lambda - T_6)(\lambda^2 - T_1\lambda - T_2T_4) = 0.$$

The Jacobian matrix $J\,(T_x, T_r, \alpha)$ has an eigenvalue $\lambda_1 = T_6$. We let λ_2 and λ_3 be the two eigenvalues different from λ_1. According to the properties of eigenvalues, we

Table A. Stability conditions for all equilibrium points of system (A4).

**Equilibrium** (x, r, c)	**Stability condition**
$(0, 0, \alpha)$	Stable if $\mu_1 < (\alpha - \beta)\mu_3$ and $\theta_1 > \theta_4$
$(0, 1, \alpha)$	Stable if $\mu_1 > (\alpha - \beta)\mu_3$
$(0, 0, \beta)$, $(0, 1, \beta)$, $(1, 0, \alpha)$ $(1, 0, \beta)$, $(1, 1, \alpha)$, $(1, 1, \beta)$	Unstable
$(x_{1t}, 1, \alpha)$	Unstable
$(x_{2t}, 1, \alpha)$	Stable if $x_{2t} < \min\left\{\frac{\mu_1 - \mu_3(\alpha-\beta)}{\mu_1+\mu_2}, \frac{\theta_1+\theta_3}{\theta_1+\theta_2}\right\}$
$(x_{1b}, 1, \beta)$	Unstable
$(x_{2b}, 1, \beta)$	Stable if $\frac{\theta_1+\theta_3}{\theta_1+\theta_2} < x_{2b} < \frac{\mu_1+\mu_4(\alpha-\beta)}{\mu_1+\mu_2}$
$(0, K_r, K_c)$	Neutrally stable
$(R_x, 1, R_c)$	Unstable
$(1, F_r, F_c)$	Unstable
(T_x, T_r, α)	Stable if $T_1 < 0$ and $T_6 < 0$
(B_x, B_r, β)	Stable if $B_1 < 0$ and $B_6 < 0$

know that $\lambda_2 + \lambda_3 = T_1$ and $\lambda_2\lambda_3 = -T_2T_4$. Combining the conditions $T_2 > 0$ and $T_4 < 0$, we conclude that the surface equilibrium point (T_x, T_r, α) is stable when $T_1 < 0$ and $T_6 < 0$.

When the surface equilibrium point (B_x, B_r, β) exists, the characteristic polynomial of $J(B_x, B_r, \beta)$ can be expressed as

$$(\lambda - B_6)(\lambda^2 - B_1\lambda - B_2B_4) = 0.$$

Applying the same analysis as for the equilibrium point (T_x, T_r, α), we conclude that the surface equilibrium point (B_x, B_r, β) is stable when $B_1 < 0$ and $B_6 < 0$.

Numerical simulations confirm the diverse dynamical regimes predicted by the theoretical analysis (Fig F). As shown in Fig Fa and Fb, a family of periodic closed orbits exists on the plane $x = 0$. In Fig Fc, the coupled system exhibits bistability between the surface equilibrium (T_x, T_r, α) and the tragic corner equilibrium $(0, 1, \alpha)$; the former corresponds to a state of moderate cooperation, with a fraction T_x of cooperators sustaining cooperation at the maximal cost α under an intermediate risk level T_r. A different bistable regime is observed in Fig Ff, where the low cost surface equilibrium (B_x, B_r, β) becomes stable alongside $(0, 1, \alpha)$, describing cooperation (fraction B_x) maintained at the minimal cost β under intermediate risk B_r (Fig Fh). In Fig Fi, we observe a bistable outcome between $(0, 0, \alpha)$ and (B_x, B_r, β). The former represents a risk-free state in which no individual pays the highest cost (Fig Fj), whereas the latter corresponds to a state where the risk level is fixed at $r = B_r$ and most individuals cooperate by paying the minimal cost β (Fig Fk).

Furthermore, when an unstable interior equilibrium exists, the system exhibits additional dynamical behaviors: trajectories converge either to the family of closed orbits on $x = 0$ (Fig Fn) or to (B_x, B_r, β) (Fig Fm). Fig Fo presents a tristable regime. Depending on initial conditions, trajectories converge to the corner equilibrium $(0, 1, \alpha)$ (Fig Fp), the surface equilibrium (T_x, T_r, α), or the surface equilibrium (B_x, B_r, β) (Fig Fq), highlighting the role of initial conditions. Due to the analytical complexity, we restrict attention to numerical results for interior equilibria. Fig Fl and Fo provide representative examples in which an interior equilibrium (x^*, r^*, c^*) exists but is unstable.

For clarity, the stability conditions for all equilibrium points of Model 2 are summarized in Table A.

We explore how the feedback speeds ε_1 and ε_2 affect the dynamics near the stable surface equilibrium (B_x, B_r, β) of Eq. (A4) (Fig G). The top row shows the effect of

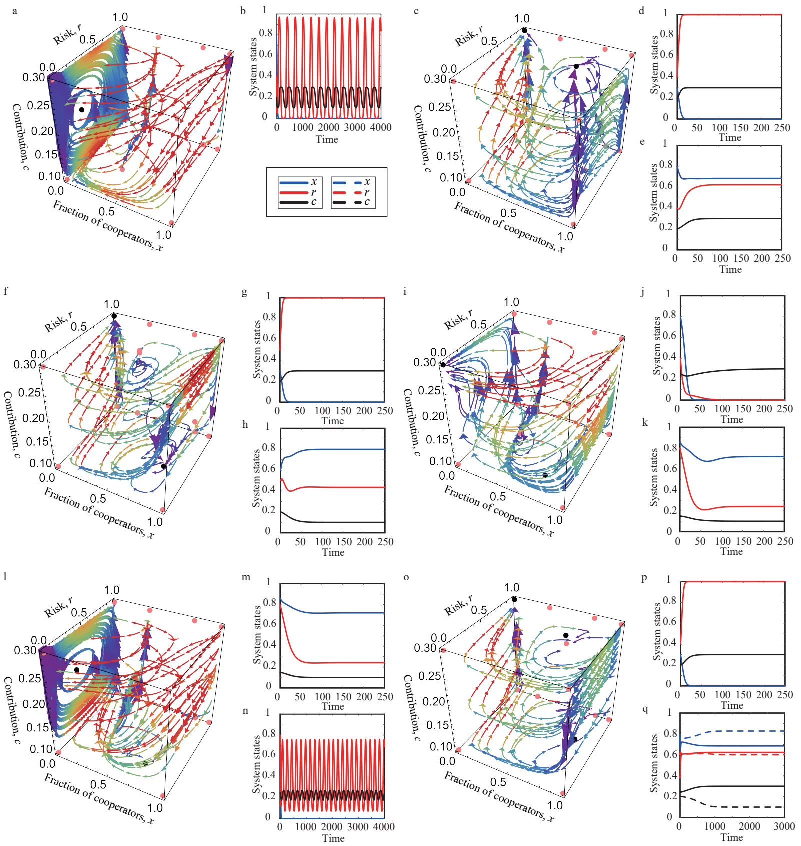

Fig F. Replicator dynamics of the coupled system exhibiting either a neutrally stable surface equilibrium or stable surface equilibria. Panels a, c, f, i, l, and o show the phase flow in $x-r-c$ space. Panels b, d, e, g, h, j, k, m, n, p, and q present the time evolution of coevolving variables. Parameters are $\mu_1 = 0.01$, $\mu_2 = 0.3$, $\mu_3 = 0.5$, $\mu_4 = 0.5$, $\theta_1 = 0.2$, $\theta_2 = 0.6$, $\theta_3 = 0.5$, $\theta_4 = 0.5$ in panels a and b; $\mu_1 = 0.5$, $\mu_2 = 0.2$, $\mu_3 = 0.1$, $\mu_4 = 0.4$, $\theta_1 = 0.6$, $\theta_2 = 0.2$, $\theta_3 = 0.8$, $\theta_4 = 0.2$ in panels c, d, and e; $\mu_1 = 0.7$, $\mu_2 = 0.3$, $\mu_3 = 1$, $\mu_4 = 0.5$, $\theta_1 = 0.5$, $\theta_2 = 0.5$, $\theta_3 = 0.35$, $\theta_4 = 0.4$ in panels f, g, and h; $\mu_1 = 0.1$, $\mu_2 = 0.15$, $\mu_3 = 1$, $\mu_4 = 0.4$, $\theta_1 = 0.3$, $\theta_2 = 0.3$, $\theta_3 = 0.25$, $\theta_4 = 0.2$ in panels i, j, and k; $\mu_1 = 0.1$, $\mu_2 = 0.15$, $\mu_3 = 1$, $\mu_4 = 0.4$, $\theta_1 = 0.1$, $\theta_2 = 0.3$, $\theta_3 = 0.25$, $\theta_4 = 0.3$ in panels l, m, and n; $\mu_1 = 0.5$, $\mu_2 = 0.2$, $\mu_3 = 0.1$, $\mu_4 = 0.4$, $\theta_1 = 0.3$, $\theta_2 = 0.2$, $\theta_3 = 0.25$, $\theta_4 = 0.2$ in panels o, p, and q. The remaining parameters $N = 8$, $M = 5$, $b = 2$, $\beta = 0.1$, and $\alpha = 0.3$ are fixed in all panels.

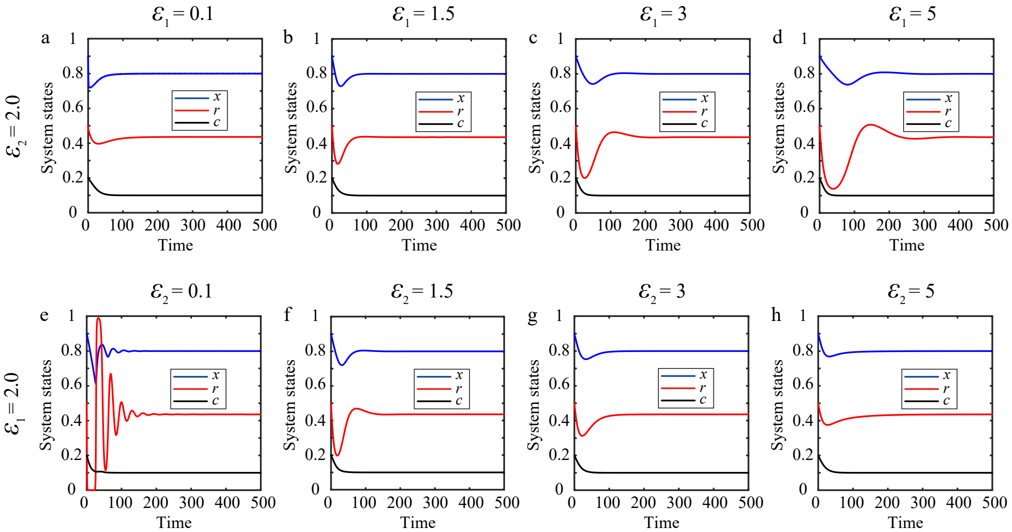

Fig G. Impact of feedback speed on the evolutionary dynamics when the system described by Eq. (A4) converges to the stable surface equilibrium point (B_x, B_r, β)**.** The top (bottom) row shows the impact of feedback speed ε_1 (ε_2) on the change of system states over time. Parameters are $N = 8$, $M = 5$, $\mu_1 = 0.7$, $\mu_2 = 0.3$, $\mu_3 = 1$, $\mu_4 = 0.5$, $\theta_1 = 0.5$, $\theta_2 = 0.5$, $\theta_3 = 0.35$, $\theta_4 = 0.4$, $b = 2$, $\beta = 0.1$, and $\alpha = 0.3$ in all panels.

varying ε_1, and the bottom row shows the effect of varying ε_2. The results indicate that variations in either feedback speed do not affect the stability of the equilibrium; rather, they only modify the rate of convergence toward the stable state.

Finally, we analyze how the feedback strengths μ_1–μ_4 shape the basins of attraction for stable equilibria in Eq. (A4) (Fig H). Varying μ_1 induces a sequence of stability transitions. For very small μ_1, only the corner equilibrium $(0, 0, \alpha)$ is stable. As μ_1 increases, $(0, 0, \alpha)$ loses stability, while (B_x, B_r, β) and $(0, 1, \alpha)$ become stable, with the latter possessing a larger basin of attraction. At intermediate values of μ_1, the system becomes tristable among (B_x, B_r, β), $(0, 1, \alpha)$, and (T_x, T_r, α). For sufficiently large μ_1, the system is bistable between $(0, 1, \alpha)$ and the edge equilibrium $(x_{2t}, 1, \alpha)$, with the latter dominating in terms of basin size (Fig Ha). Turning to the other parameters, increasing the cooperation-to-risk damping coefficient μ_2 systematically enlarges the basin of attraction of $(0, 1, \alpha)$ (Fig Hb). A stronger cost-to-risk mitigation effect (μ_3) also favors $(0, 1, \alpha)$, whose basin of attraction grows monotonically, whereas the basin of (B_x, B_r, β) first expands and then contracts (Fig Hc). For the risk-amplifying effect associated with lower cooperation cost (μ_4), small values lead to a tristable regime in which the basins of attraction decrease in the order $(0, 1, \alpha) > (B_x, B_r, \beta) > (T_x, T_r, \alpha)$. As μ_4 increases, (B_x, B_r, β) becomes destabilized, and eventually the basin of (T_x, T_r, α) exceeds that of $(0, 1, \alpha)$ (Fig Hd). Importantly, a key focus is the evolution of the attraction domain for the surface equilibrium (B_x, B_r, β). This state represents the stable coexistence of cooperators and defectors, where cooperators sustain cooperation at the minimal cost β while maintaining a constant risk level B_r. Our numerical results indicate that appropriately adjusting the system's feedback coefficients can significantly enhance the accessibility and stability of this desirable cooperative regime.

We further investigate the impact of the cost-feedback coefficients θ_1–θ_4 on the basins of attraction for each stable equilibrium (Fig He-h). We find that the basin of attraction of the equilibrium $(0, 1, \alpha)$ increases gradually with parameters θ_1 and θ_3, but decreases gradually with θ_2 and θ_4. For the equilibrium (T_x, T_r, α), its basin of

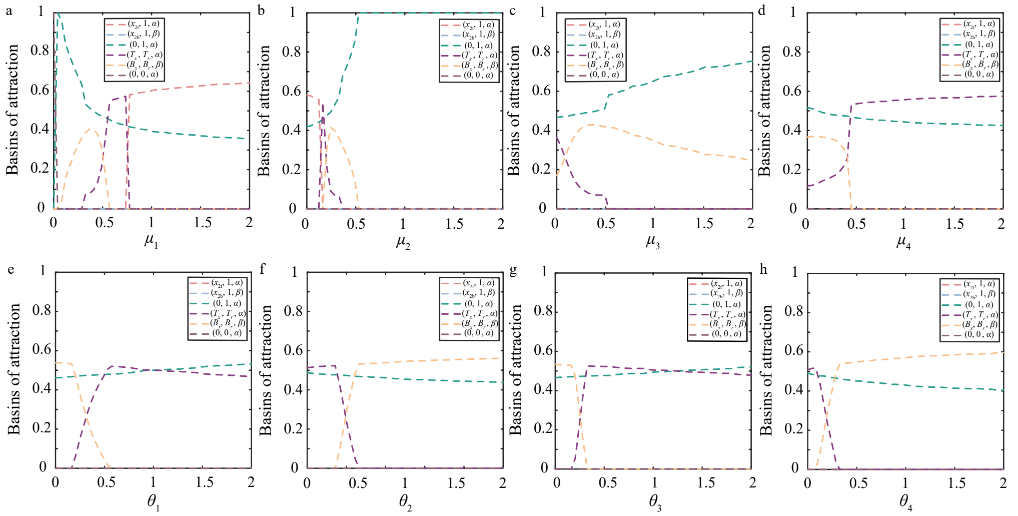

Fig H. The influence of model parameters on the attraction domains of stable equilibrium points in the coupled system Eq. (A4). μ_1 and μ_2 characterize the coefficients of the effects of changes in the fractions of defectors and cooperators on collective risk, while μ_3 and μ_4 represent the coefficients associated with increases and decreases in cooperation cost in relation to risk. θ_1 and θ_2 denote the coefficients for the impact of changes in the fractions of defectors and cooperators on cooperation cost, whereas θ_3 and θ_4 represent the coefficients for the effects of increases and decreases in risk on cooperation cost. Variations in parameter values may influence the evolutionary dynamics of the system. Parameters are $\mu_2 = 0.2$, $\mu_3 = 0.1$, $\mu_4 = 0.4$, $\theta_1 = 0.3$, $\theta_2 = 0.2$, $\theta_3 = 0.25$, $\theta_4 = 0.2$ in panel a; $\mu_1 = 0.5$, $\mu_3 = 0.1$, $\mu_4 = 0.4$, $\theta_1 = 0.3$, $\theta_2 = 0.2$, $\theta_3 = 0.25$, $\theta_4 = 0.2$ in panel b; $\mu_1 = 0.5$, $\mu_2 = 0.2$, $\mu_4 = 0.4$, $\theta_1 = 0.3$, $\theta_2 = 0.2$, $\theta_3 = 0.25$, $\theta_4 = 0.2$ in panel c; $\mu_1 = 0.5$, $\mu_2 = 0.2$, $\mu_3 = 0.1$, $\theta_1 = 0.3$, $\theta_2 = 0.2$, $\theta_3 = 0.25$, $\theta_4 = 0.2$ in panel d; $\mu_1 = 0.5$, $\mu_2 = 0.2$, $\mu_3 = 0.1$, $\mu_4 = 0.4$, $\theta_2 = 0.2$, $\theta_3 = 0.25$, $\theta_4 = 0.2$ in panel e; $\mu_1 = 0.5$, $\mu_2 = 0.2$, $\mu_3 = 0.1$, $\mu_4 = 0.4$, $\theta_1 = 0.3$, $\theta_3 = 0.25$, $\theta_4 = 0.2$ in panel f; $\mu_1 = 0.5$, $\mu_2 = 0.2$, $\mu_3 = 0.1$, $\mu_4 = 0.4$, $\theta_1 = 0.3$, $\theta_2 = 0.2$, $\theta_4 = 0.2$ in panel g; $\mu_1 = 0.5$, $\mu_2 = 0.2$, $\mu_3 = 0.1$, $\mu_4 = 0.4$, $\theta_1 = 0.3$, $\theta_2 = 0.2$, $\theta_3 = 0.25$ in panel h. The remaining parameters $N = 8$, $M = 5$, $b = 2$, $\beta = 0.1$, and $\alpha = 0.3$ are fixed in all panels.

attraction expands initially and then contracts as θ_1 or θ_3 rises; in contrast, with increasing θ_2 or θ_4, it first increases slowly and then declines rapidly. Notably, the basin of attraction of (B_x, B_r, β) remains nearly constant at first under increasing θ_1 or θ_3, before dropping sharply to zero. Conversely, with rising θ_2 or θ_4, it grows rapidly initially and then continues to increase at a slower rate. It is particularly noteworthy that when θ_2 and θ_4 are sufficiently high, the basin of attraction of (B_x, B_r, β) surpasses that of $(0, 1, \alpha)$.

References

1. Ma Z, Zhou Y. Characterization and Stability Methods for Ordinary Differential Equations. Beijing: Beijing Science Press; 2001.

2. Khalil H. Nonlinear Systems. 3rd ed. Upper Saddle River, NJ: Prentice Hall; 2002.

3. Betz K, Fu F, Masuda N. Evolutionary game dynamics with environmental feedback in a network with two communities. Bulletin of Mathematical Biology. 2024;86(7):84.
